# Strontium-Substituted Dicalcium Silicate Bone Cements with Enhanced Osteogenesis Potential for Orthopaedic Applications

**DOI:** 10.3390/ma12142276

**Published:** 2019-07-15

**Authors:** Wenjuan Liu, Zhiguang Huan, Min Xing, Tian Tian, Wei Xia, Chengtie Wu, Zhihua Zhou, Jiang Chang

**Affiliations:** 1 School of Materials Science and Engineering, Hunan University of Science and Technology, Xiangtan 411201, China; 2 Division of Applied Materials Science, Department of Engineering Sciences, The Ångström Laboratory, Uppsala University, SE-751 21 Uppsala, Sweden; 3 Hunan Provincial Key Laboratory of Controllable Preparation and Functional Application of Fine Polymers, Hunan University of Science and Technology, Xiangtan 411201, China; 4 State Key Laboratory of High Performance Ceramics and Superfine Microstructure, Shanghai Institute of Ceramics, Chinese Academy of Sciences, 1295 Dingxi Road, Shanghai 200050, China; 5 School of Chemistry and Chemical Engineering, Hunan University of Science and Technology, Xiangtan 411201, China

**Keywords:** dicalcium silicate, strontium, bone cement, orthopaedics, stomatology, bone regeneration

## Abstract

Incorporating Sr element in biomaterials for bone implants is an effective way to improve their biological performance, as Sr element has been proved to enhance bone regeneration and depress bone resorption activity. In the present study, we developed a Sr-incorporated dicalcium silicate (C2S) bone cement as a potential candidate for bioactive self-setting bone cement in orthopaedics and stomatology. The Sr-C2S powders containing 0.3–6.8% Sr in molar ratio were prepared by means of chemical co-precipitation, and the results of XRD analysis indicated the incorporation of Sr element into the lattice of C2S. Sr-C2S bone cements, as prepared by mixing the powders with water, have a final setting time of 570 to 594 min, and compressive strength higher than that of C2S bone cement within certain incorporation range. The Sr-C2S bone cements possessed good in vitro bioactivity by inducing apatite formation in simulated body fluid (SBF) within 7 days. Moreover, the proliferation activity of human bone marrow mesenchymal stem cells (hBMSCs) with Sr-C2S bone cements was significantly higher than that with C2S bone cement, and the alkaline phosphatase (ALP) activity of hBMSCs was also enhanced with addition of Sr element in Sr-C2S groups. The Sr-C2S might therefore be a bioactive self-setting material with enhanced biological performance and holds the prospect for application in the bone regeneration area.

## 1. Introduction

Dicalcium silicate (Ca_2_SiO_4_, C2S) cement has been developed as a new type of bone cement for the restoration of osseous and dental defect [1,2]. As an important constituent of Portland cement, C2S possesses hydraulic property and can react with water or aqueous solution to form calcium silicate hydrate (C-S-H) as main hydration product, which contributes to the self-setting property and spontaneously increasing strength of the material. The C2S paste can be implanted at the defect site through injection, thus avoiding large surgery openings. Similar to other silicate-based bioactive materials, e.g., calcium silicate and bioactive glass, C2S can induce bone-like apatite mineralization in simulated body fluid [1,2]. Moreover, several studies have demonstrated that C2S could support the proliferation and differentiation of osteo-related cells, due to the release of Ca and Si [3,4,5,6,7]. However, it may be not enough to repair bone tissues with large defect size and poor osteogenic ability through C2S alone, as previous studies indicated that calcium silicate show limited effect on restoration of osteoporotic bone tissue [8]. Thus, it is necessary to find solutions to improve the osteogenic ability of C2S, especially on occasions of repairing bone tissue with poor osteogenic ability.

Sr is the second main group element in the periodic table, and is well-known for its important role in bone regeneration. It has been well established both in research and clinical application that Sr element could significantly promote bone regeneration and inhibit bone resorption [9,10,11,12]. The favorable effect of Sr on bone metabolism leads to a great interest in the study of Sr-incorporated materials, such as Sr-substituted hydroxyapatite (Sr-HA) [13,14], bioactive glass (Sr-BG) [15,16] and calcium silicate (Sr-CS) [8,17]. Previous studies suggested that silicate bioceramics incorporated with Sr element possessed better performance by enhancing bone regeneration and suppressing bone resorption, which indicated that Sr could improve osteogenic activity of silicate bioceramics. However, to our knowledge, the effect of Sr in silicate-based bone cement has not been well investigated. 

C2S powder can be synthesized by different methods according to literature report, including solid state sintering [18] and the sol-gel method [2,7]. Solid state sintering with CaO and SiO_2_ as raw materials tends to leave CaO residues, and its sintering temperature is higher than that of the so-gel method. The sol-gel method can also cause CaO impurities. In the present study, we propose a chemical co-precipitation method for the preparation of C2S, which is expected to produce fine and pure C2S powders at relatively low sintering temperature. 

Considering the self-setting and biological properties of C2S and the osteo-stimulation properties of Sr element, the incorporation of Sr into C2S may lead to the development of self-setting bone cement with enhanced bone regeneration ability. Therefore, the aim of this study is to synthesize Sr-incorporated C2S (Sr-C2S) and investigate the effect of Sr incorporation on the self-setting and biological properties of the bone cements. The self-setting properties of Sr-C2S bone cements were evaluated in terms of their setting times and compressive strength after hydration. The apatite mineralization ability, cytocompatibility and ALP differentiation activity of Sr-C2S bone cements were investigated and compared with those of C2S bone cement in order to confirm the biological benefit of Sr-incorporation.

## 2. Materials and Methods 

### 2.1. Preparation and Characterization of C2S and Sr-C2S Powders

C2S powder was prepared by the chemical co-precipitation method. To prepare 0.1 mol C2S powder, 1 mol·L^−1^ Ca(NO_3_)_2_·4H_2_O (200 mL), Na_2_SiO_3_·9H_2_O (100 mL) and Na_2_CO_3_ (100 mL) solutions were prepared. Among the raw materials, Na_2_CO_3_ was used as a co-precipitate reagent to make half the amount of Ca(NO_3_)_2_·4H_2_O precipitate, and the CO_3_^2−^ was removed during sintering. Na_2_SiO_3_·9H_2_O and Na_2_CO_3_ solutions were added drop by drop into Ca(NO_3_)_2_·4H_2_O solution with stirring. During dropping, the pH of the mixture solution was adjusted to 12 with ammonia solution. Subsequently, the mixture was continuously stirred for 24 h, and was then filtered. The precipitant was collected and washed with de-ionized water and ethanol for three times, respectively. After washing, the precipitant was dried in an oven (60 °C) for 24 h and then sintered at 1000 °C for 3 h. The sintered powder was then ground and sieved through a 200-mesh sift.

The preparation of Sr-C2S powder was similar to that of C2S powder as described above, except that the raw reagent of Ca(NO_3_)_2_·4H_2_O was partly substituted by Sr(NO_3_)_2_. A series of Sr-C2S with different amounts of Sr was prepared by substitution of 1, 5 and 10 mol.% Ca(NO_3_)_2_·4H_2_O by Sr(NO_3_)_2_. The amounts required for the preparation of 0.1 mol C2S or Sr-C2S powder are represented in Table 1.

The phase composition and morphology of C2S and Sr-C2S powders were characterized by X-ray diffractometer (XRD, Geigerflex, Rigaku Co., Tokyo, Japan) with Cu (Kα) radiation and scanning electron microscope (SEM, Hitachi S4800, Hitachi Ltd., Tokyo, Japan), respectively. To investigate the element distribution of Sr-C2S powders, EDS element mapping analysis (Ultim^®^ Extreme, Oxford Instruments, Abingdon, Oxfordshire, England) was conducted on Sr10-C2S powder as a representative sample. The content of each element in C2S and Sr-C2S powders was tested by X-ray fluorescence spectrometer (XRF, AXIOS, PANalytical B. V., Almelo, the Netherlands).

### 2.2. Characterization of the C2S and Sr-C2S Bone Cement

#### 2.2.1. Phase Compositions and Microstructure of Hydrated Cements

The C2S and Sr-C2S bone cements were prepared by mixing the powders with de-ionized water, and the liquid to powder ratio (LPR) was set to 0.5 mL·g^−1^. The mixture was stirred to homogeneous paste and filled into a mold with cylindrical hole (φ = 6 mm, h = 12 mm). Then, the cylindrical sample was cured in a constant-temperature shaking water bath at 37 °C, de-molded after 1 day and kept curing for 13 days. The phase compositions of different cements were characterized by XRD, and morphology of the cross-section of the cements were observed by SEM (JSM-6380LV, JEOL, Ltd., Akishima, Tokyo, Japan).

#### 2.2.2. Setting Time

The setting time of C2S and Sr-C2S bone cements was tested according to ISO-9597-1989E [19]. The cement powder and deionized water were mixed homogeneously to cement paste, and the liquid-to-powder ratio (LPR) was 0.5 mL·g^−1^. Then, the paste was poured into a cylindroid container, cured in a constant-temperature shaking water bath at 37 °C, and then the setting time was tested using a Vicat apparatus. The initial setting time of the cement was defined as the time from de-ionized water was added into the cement powder, to the time when the light needle kept 5 mm distance to the bottom of the container, and final setting time was defined as the time when the heavy needle failed to make evident indentations on the surface of the paste. Three parallel samples were used for each test.

#### 2.2.3. Compressive Strength of the Bone Cements

The cement paste of C2S and Sr-C2S was poured into a cylindroid mold 12 mm in height and 6 mm in internal diameter, and demolded after 24 h of reservation. The demolded cement samples were then further cured in a constant-temperature shaking water bath for 13 days in a water bath at 37 °C, and then tested on a universal testing machine (AG-I, SHIMADZU Corporation, Kyoto, Japan), at a loading speed of 0.5 mm·min^−1^. Three parallel samples were used for each test.

### 2.3. Apatite Mineralization

To evaluate apatite mineralization ability, C2S and Sr-C2S cements were made into disks (φ = 6 mm, h = 2 mm), and each disc was soaked in 9.4 mL simulated body fluid (SBF, See Table 2 for composition) solution, in a polyethylene bottle placed in a constant-temperature water bath at 37 °C for 7 days. SBF solution was prepared according to the method reported by Kokubo [20], and was refreshed every 2 days. After soaking for 7 days, the samples were taken out and gently rinsed with de-ionized water and dried in the air for 24 h. The as-prepared samples were analyzed by Fourier Transform Infrared Spectroscopy (FTIR; Thermo Nicolet nexus-IR spectrometer; Thermo Fisher Scientific, Waltham, MA, USA), XRD and SEM, respectively. For FTIR test, KBr discs with sample powder were prepared and tested in the range of 4000–400 cm^−1^, and the spectroscopy was subtracted by that of the blank KBr disk.

### 2.4. In Vitro Cytocompatibility Test

The effect of C2S and Sr-C2S cements on the proliferation of human bone marrow mesenchymal stem cells (hBMSCs, Cyagen Biosciences Co. Ltd., Taicang, Jiangsu, China, passage 5) was evaluated by Cell Counting Kit (CCK)-8 assay (CCK-8, Beyotime technology Co. Ltd., Shanghai, China ) [21]. C2S and Sr-C2S cements were made into discs (φ = 6 mm, h = 2 mm), and each disc was combined with 2 mL culture medium. After immersion in the medium for 24 h, the extracts were collected and centrifugated at 4000 rpm for 10 min. The obtained extract was diluted with culture medium to different concentrations from 100 mg·mL^−1^ to 3.125 mg·mL^−1^. The hBMSCs (1 × 10^3^ cells/well) were seeded on the 96-well plates and cultured in a humidified incubator containing 5% CO_2_ at 37 °C. After 12 h, the culture medium was replaced by C2S and Sr-C2S extract at different concentrations. Then, after cultured for 1 and 5 days, the cells in different groups were tested with a Cell Counting Kit (CCK)-8 assay (Beyotime) according to the manufacturer’s instructions. Briefly, after culturing for 1 and 5 days, the culture medium was removed and replaced with fresh medium containing CCK-8 (10:1), and the cells were further cultured at 37 °C in an incubator for 2 h. The absorbance of the reaction product was measured with an enzyme-linked immunoadsorbent assay microplate reader (Synergy 2, Bio-TEK Co. Ltd., Winooski, VT, USA) at 450 nm wavelength. The optical density (OD) values were used to represent the number and metabolic activity of hBMSCs. Three parallel samples were used for each test. 

### 2.5. ALP Activity Assay

The ALP activity assay was conducted according to previous studies [21]. The hBMSCs were cultured in medium containing C2S and Sr-C2S extracts at 100 mg·mL^−1^, and medium without extracts, respectively. ALP activity was tested after culturing for 10 days and pNPP substrates (p-nitrophenyl phosphate substrates, Sigma-Aldrich, St. Louis, MO, USA) was used according to the manufacturer’s protocol. Briefly, the cells were washed three times with PBS to remove the residual medium, and then lysed in 200 mL of 10 mM Tris–HCl buffer containing 0.1% TritonX-100 for 10 min at 20 °C. The cell lysates were then transferred into Eppendorfs microcentrifuge tube and centrifuged (10000 rpm, 10 min) at 4 °C. The obtained supernatant (100 mL) was transferred to 96-well plates and mixed with 200 mL pNPP solution. The mixture was kept in darkness for 30 min at room temperature and then tested to obtain absorbance at 405 nm with a micro-plate reader. Data were normalized to the total cell protein content as measured by a Pierce^®^ BCA Protein Assay Kit (Thermo scientific, Waltham, MA, USA). The relative ALP activity was denoted by absorbance (OD value) per milligram of total cellular proteins. Three parallel samples were used for each test.

### 2.6. Statistical Analysis

The data were denoted as mean ± standard deviation (SD), and analyzed using one-way ANOVA with Post Hoc analysis. If the obtained *p* < 0.05, the two groups of data were considered significantly different.

## 3. Results and discussion

### 3.1. Characterization of C2S and Sr-C2S Powders

**Figure 1:**
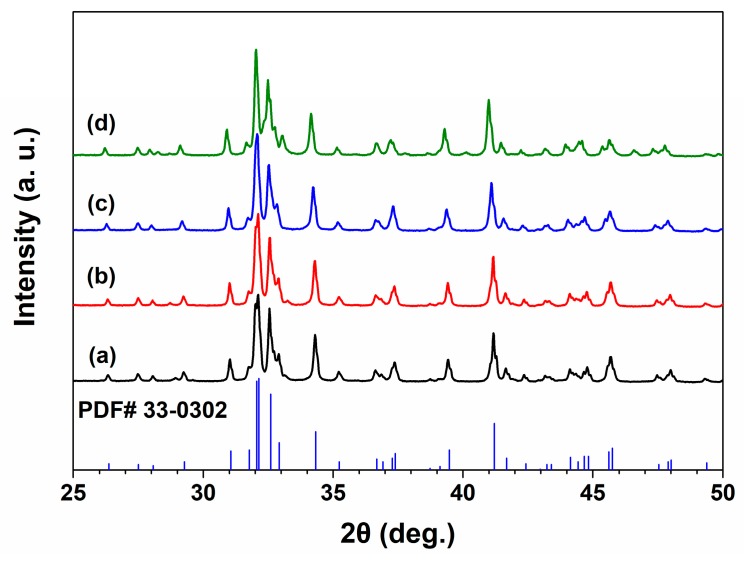
XRD patterns of C2S and Sr-C2S powders: (**a**) C2S; (**b**) Sr1-C2S; (**c**) Sr5-C2S; (**d**) Sr10-C2S. The XRD patterns of C2S and Sr-C2S powders are consistent with that of C2S standard diffraction data (PDF# 33-0302).

The XRD patterns of C2S and Sr-C2S powders, as shown in Figure 1, suggest that both C2S and Sr-C2S powders with different Sr content can be identified as pure C2S phase (ICDD PDF no. 33-0302). There was no new diffraction peak appearing in the XRD patterns of Sr-C2S as compared with that of C2S. The similarity between the XRD patterns of C2S and Sr-C2S powders indicates that the phase and crystal structure of Sr-C2S is identical with C2S, and the Sr element may enter the crystal lattice of C2S by substituting part of Ca. Compared with those of C2S, the diffraction peaks of Sr-C2S shift to lower degrees as shown in Table 3. Moreover, with the increase of Sr content, the degree of the shift becomes greater. The strong double peaks around 32° gradually overlap and merge into a single peak. It is known that the radius of Sr^2+^ (1.12 Å) is significantly larger than that of Ca^2+^ (0.99 Å) [22], resulting in a larger inter-planar distance in Sr-C2S crystal lattice; thus, the diffraction peaks of Sr-C2S shift to lower degrees in their XRD patterns. The larger lattice parameters caused by dropping of Sr were reported by an early study by Fukuda et al., in which they found lattice parameters as well as the volume of unit cell increased with the incremental content of Sr [23]. Previous studies on Sr-substituted hydroxyapatite and calcium phosphate cement also demonstrated that the substitution of Ca by Sr led to left-shift of diffraction peaks [24,25]. Therefore, it can be inferred that the Sr-C2S are formed by in situ Sr-substitution in the site of Ca after calcination. The in situ Sr-substitution of Ca can be interpreted as the formation of C2S and strontium silicate solid solution, and thus can be explained by the theory regarding solid solutions. The difference between radius of Ca^2+^ (114 pm) and radius of Sr^2+^ (132 pm) is 13.64%, below the critical value (15%) for formation of replacement solid solution. Therefore, Sr is merged in the lattice structure of C2S rather than forming its own crystalline structure of strontium silicate, which is favorable for decreasing the Gibbs free energy of the system. Although many studies have been conducted to introduce Sr element in bone regeneration materials, they are mainly focused on calcium phosphate systems and some calcium silicate ceramics [8,24,25]. 

Recently, Huang et al. reported a Sr-incorporated calcium silicate bone cement [18]. However, the synthesis method and the final sintering products are quite different in the present study, compared with the literature report. The chemical precipitation method with a lower sintering temperature (1000 °C) was used in the present study, compared with the solid-state sintering method with a sintering temperature of 1400 °C in Huang’s study. Moreover, the different sintering method leads to significant difference in the phases of final products. The cement powders in the present study are one homogeneous phase, compared with a mixture of different compounds in the article. These differences also lead to different properties of the cements, for example, the setting time, which will be discussed in the corresponding section. To the best of our knowledge, the present study is the first report on in situ doping of Sr in to C2S as bone cement by the chemical co-precipitation method. 

To quantify the amount of Sr element in Sr-C2S powders, XRF analysis was conducted on the powders and the result are shown in Table 4. It can be seen that Sr exists in each group of the Sr-C2S powders and its content increases in the order of Sr1-C2S, Sr5-C2S and Sr10-C2S. However, it was noted that the actual content (both the molar and weight percentage) of Sr element in all samples was lower than the theoretical content. These results may be caused by the loss of Sr during the procedures of washing and filtration. The adjustable Sr content provides a broad option for the composition of materials.

**Figure 2:**
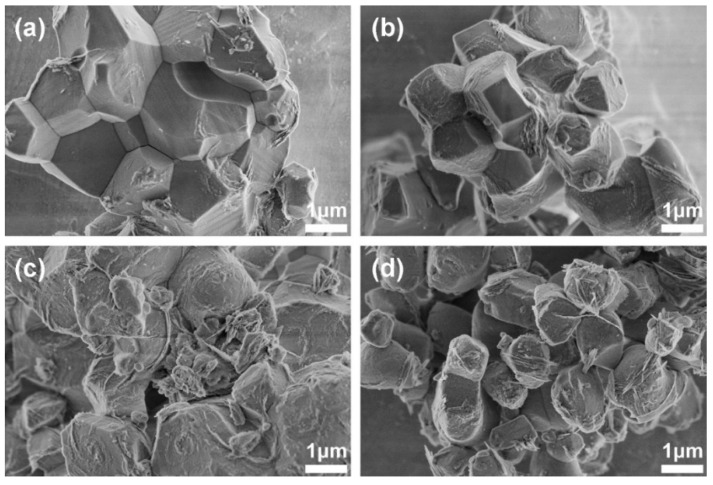
SEM images of C2S and Sr-C2S powders: (**a**) C2S; (**b**) Sr1-C2S; (**c**) Sr5-C2S; (**d**) Sr10-C2S.

**Figure 3:**
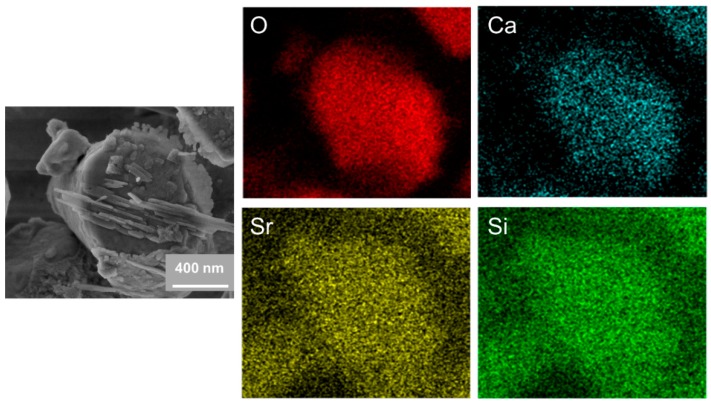
SEM images of Sr10-C2S particles and corresponding EDS element mapping images.

The SEM images of the C2S and Sr-C2S powders are shown in Figure 2. The particles of C2S and Sr-C2S by chemical co-precipitation method in the present study are around 1~4 micrometers and aggregate together to form larger agglomerations, which are comparable with those made by the sol-gel method [7]. The incorporation of Sr into C2S has no obvious influence on the morphology and size of the particles. The EDS element mapping images of Ca, Sr, Si, O in Sr-C2S as demonstrated in Figure 3 show that the Sr element is homogenously distributed within Sr-C2S particles. 

### 3.2. Composition and Microstructure of the Bone Cements

**Figure 4:**
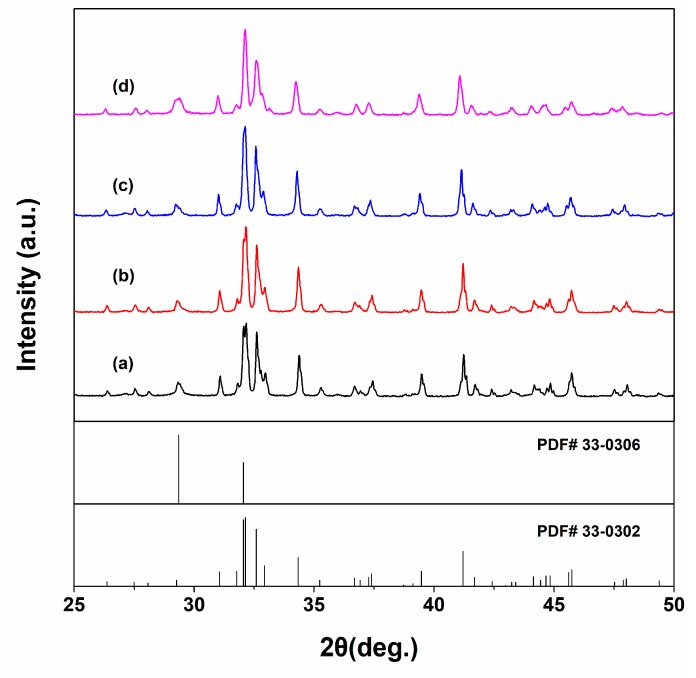
XRD patterns of C2S and Sr-C2S bone cements after curing in a constant-temperature shaking water bath at 37 °C for 14 days: (**a**) C2S; (**b**) Sr1-C2S; (**c**) Sr5-C2S; (**d**) Sr10-C2S. PDF# 33-0302 and 33-0306 are standard diffraction data of C2S and CSH, respectively. The XRD patterns of C2S and Sr-C2S bone cements are consistent with that of C2S (PDF# 33-0302). The diffraction peaks of CSH (PDF# 33-0306) overlap with those of C2S.

**Figure 5:**
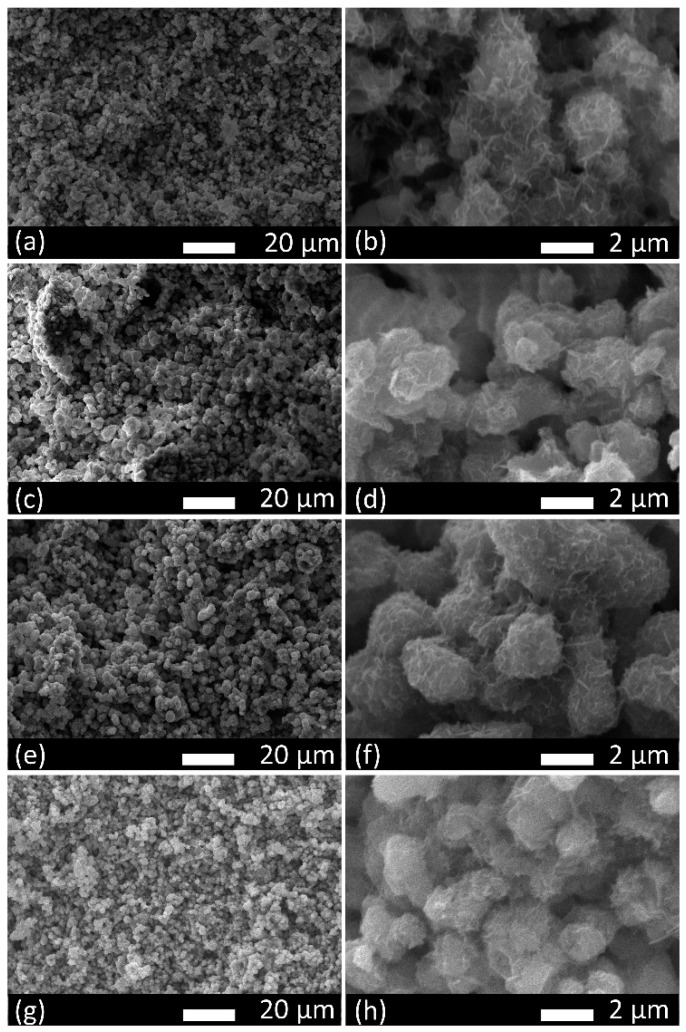
SEM images of C2S and Sr-C2S cements after curing in a constant-temperature shaking water bath at 37 °C for 14 days: (**a**,**b**) C2S; (**c**,**d**) Sr1-C2S; (**e**,**f**) Sr5-C2S; (**g**,**h**) Sr10-C2S.

The XRD patterns of C2S and Sr-C2S bone cements are shown in Figure 4. The hydration product of C2S is CSH, which is a poorly crystalline phase and not distinct in the XRD patterns [26]. The microstructure of the hydrated bone cements with various content of Sr incorporation were shown in Figure 5. It can be seen that, regardless of Sr incorporation, a fibrillar surface was formed on the cement particles after hydration. The fibrillar surface is typical morphology of CSH, as the main reason for the self-setting process [26]. The present study suggests that Sr-C2S with the Sr-content up to 6.427 wt.% keeps the self-setting property of C2S bone cement, which meets our expectation for a Sr-containing C2S self-setting material.

### 3.3. Setting Time and Compressive Strength of the Bone Cements

**Figure 6:**
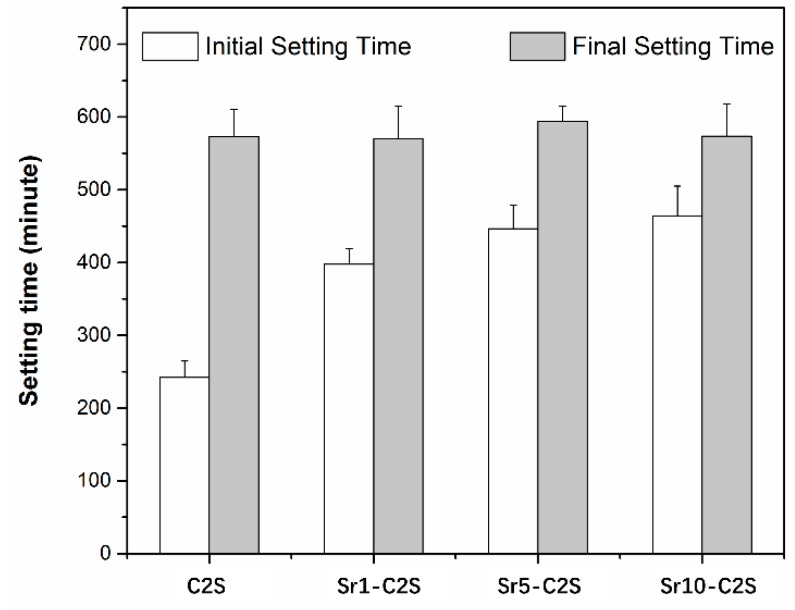
Initial and final setting time of C2S and Sr-C2S bone cement. The initial setting time increases with increasing content of Sr.

The initial setting time of the bone cement increases with the incremental addition of Sr element, ranging from 242 min for C2S to 464 min for Sr10-C2S, as shown in Figure 6. The final setting time shows no obvious difference between C2S and Sr-C2S bone cements. The setting process of C2S involves a hydraulic reaction and the establishment of a gel network by the hydration product, CSH. The speed for hydration process of C2S is relatively slow, resulting in a longer setting time compared with that of C3S. Previous studies on sol-gel synthesized C2S bone cement show that its initial setting time ranged from 60 to 180 min, and final setting time ranged from 220 to 370 min. The recent study on Sr-substituted calcium silicate bone cement by Huang et al. demonstrated a quite fast setting process with setting time between 11–19 min [18]. Our results show obvious longer initial and final setting times compared with previous studies, which could be caused by different preparation methods and sintering temperature. Sr element in Sr-C2S cements may have interfered with the formation of CSH gel and lead to prolonged initial setting time. Setting time is a key consideration in the application of bone cement. The Sr-C2S bone cements in the present study hold an adjustable setting time via altering their Sr content. Setting time is an important consideration in clinical application of bone cements and its ideal range can be referred to the setting time of widely used PMMA (Poly(methyl methacrylate)) bone cements, which is between approximately 7.5 and 26.5 min. The Sr-C2S bone cements in the present study have much longer setting time than clinical demands; thus, it is a problem that remains to be solved in future investigations. 

**Figure 7:**
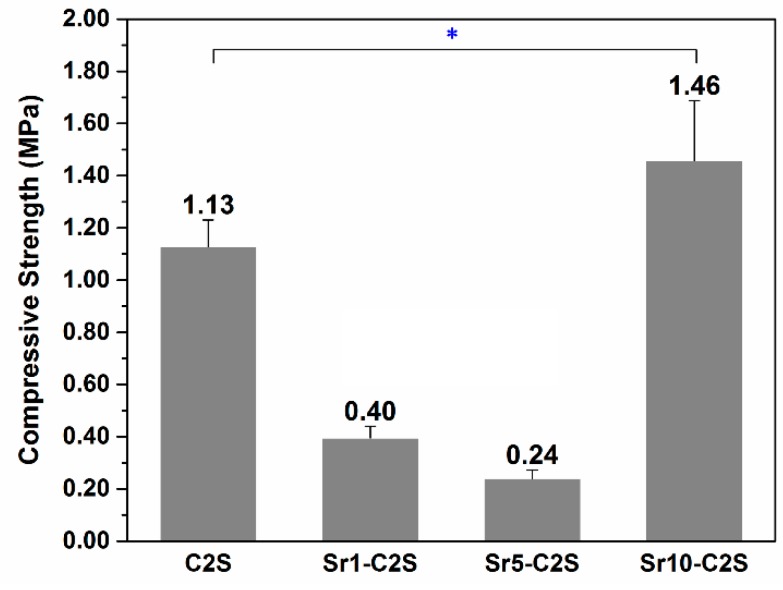
Compressive strength of C2S and Sr-C2S bone cement after curing in a constant-temperature shaking water bath at 37 °C, 100% humidity for 14 days. * denotes significant difference exists between the two groups.

The compressive strength of the C2S and Sr-C2S bone cements after hydration are shown in Figure 7. As compared with C2S bone cement, Sr1-C2S and Sr5-C2S bone cements show obvious decrease in compressive strength. However, it can be seen that the compressive strength of Sr10-C2S cement significantly increases as compared with that of C2S bone cement. The compressive strength of different Sr-C2S bone cements presents an irregular change with the content of Sr, which may be attributed to the changes of crystal lattice caused by Sr. 

### 3.4. Apatite Mineralization

**Figure 8:**
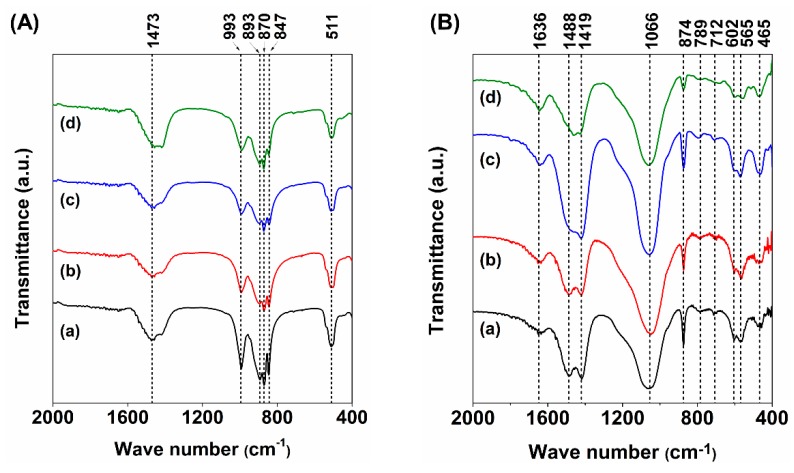
FTIR of C2S and Sr-C2S bone cement before (**A**) and after (**B**) immersion in SBF solution for 7 days: (**a**) C2S; (**b**) Sr1-C2S; (**c**) Sr5-C2S; (**d**) Sr10-C2S. The interpretation of different absorption peaks is as follows: (**A**) 1474 cm^−1^ and 870 cm^−1^—CO_3_^2−^, the absorption band between 893 cm^−1^ to 847 cm^−1^ and the absorption peaks 993 cm^−1^ and 511 cm^−1^—Si-O-Si; (B) 1636 cm^−1^ and 789 cm^−1^—H_2_O, 1488 cm^−1^, 1419 cm^−1^, 874 cm^−1^ and 712 cm^−1^—CO_3_^2−^, 465 cm^−1^—Si-O-Si, 1066 cm^−^, 602 cm^−1^ and 565 cm^−1^—PO_4_^3−^. It can be clearly seen that phosphate salts formed on the surface of the cements after immersion in SBF solution.

**Figure 9:**
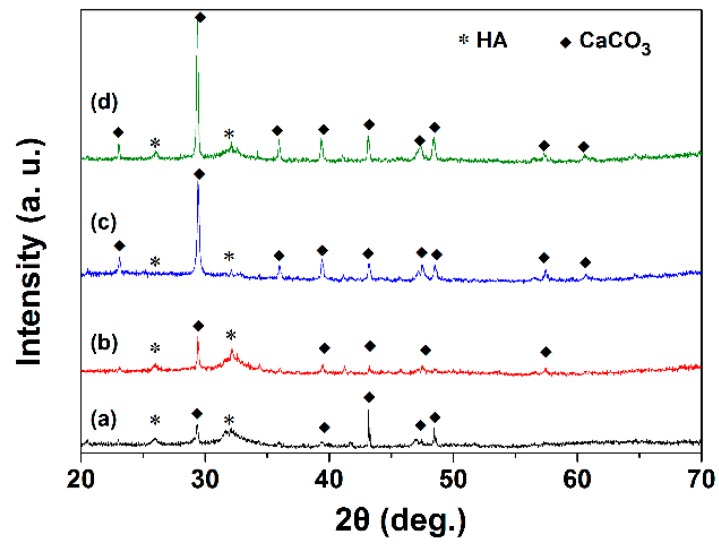
XRD patterns of C2S and Sr-C2S bone cements after immersion in SBF solution for 7 days: (**a**) C2S; (**b**) Sr1-C2S; (**c**) Sr5-C2S; (**d**) Sr10-C2S. The results suggest apatite formation on all C2S and Sr-C2S bone cements.

**Figure 10:**
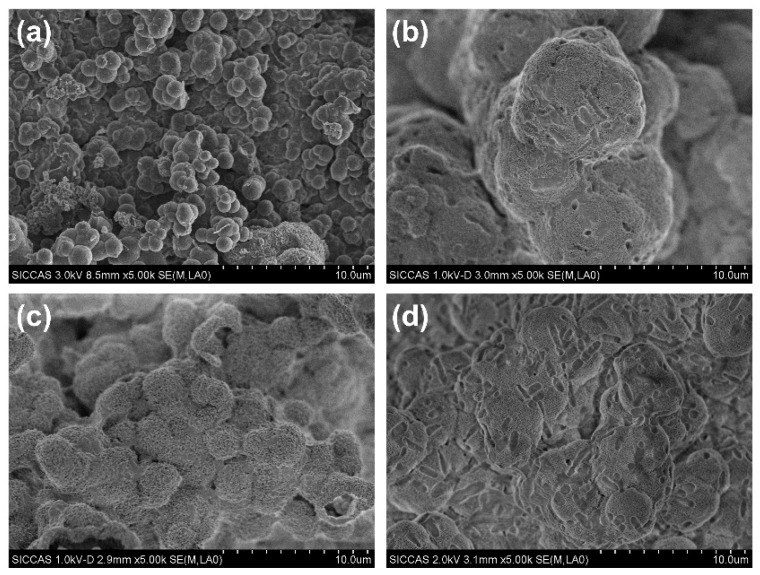
SEM images of C2S and Sr-C2S after immersion in SBF solution for 7 days: (**a**) C2S; (**b**) Sr1-C2S; (**c**) Sr5-C2S; (**d**) Sr10-C2S.

The apatite mineralization of C2S and Sr-C2S cements after immersion in SBF solution for 7 days was characterized by IR, XRD and SEM, and the results are presented in Figure 8, Figure 9 and Figure 10, respectively. In comparison with hydrated cements (Figure 8A), it can be clearly seen that PO_4_^3−^ peaks (1066, 602 and 565 cm^−1^) appear after the cements were immersed in SBF solutions. In particular, the double peaks at 602 and 565 cm^−1^ are characteristics for crystalline apatite, suggesting formation of apatite on C2S and Sr-C2S bone cements. XRD patterns show the characteristic diffraction peaks of apatite (Figure 9), which further confirmed the apatite formation ability of C2S and Sr-C2S bone cements. It can be noticed that the diffraction peaks of apatite in Sr5-C2S are weaker than the other groups, which may indicate that the crystallinity of the formed apatite is lower at this level of Sr content. SEM images demonstrated the morphology of apatite formed on C2S and Sr-C2S bone cements (Figure 10). The mineralized apatite appears to be sphere-like, with differences in sphere size and morphology among the different samples. The discrepancy in apatite morphology may be caused by the different content of Sr as well as the particle sizes. Huang et al. found that the addition of Sr could delay the speed of hydration and apatite formation, and then put forward that Sr affects the apatite formation kinetics by competitive electrostatic adsorption at the nucleation site of apatite [18]. Although in the present study the formation rate of apatite was not reflected directly, the difference in morphology and XRD peaks suggested that Sr content can affect the size, shape and crystallinity of the apatite. The apatite formed on the interface between the implanted material and bone tissue lead to firm chemical binding between them, which is favorable for bone integration and regeneration [27].

### 3.5. In Vitro Cytocompatibility

**Figure 11:**
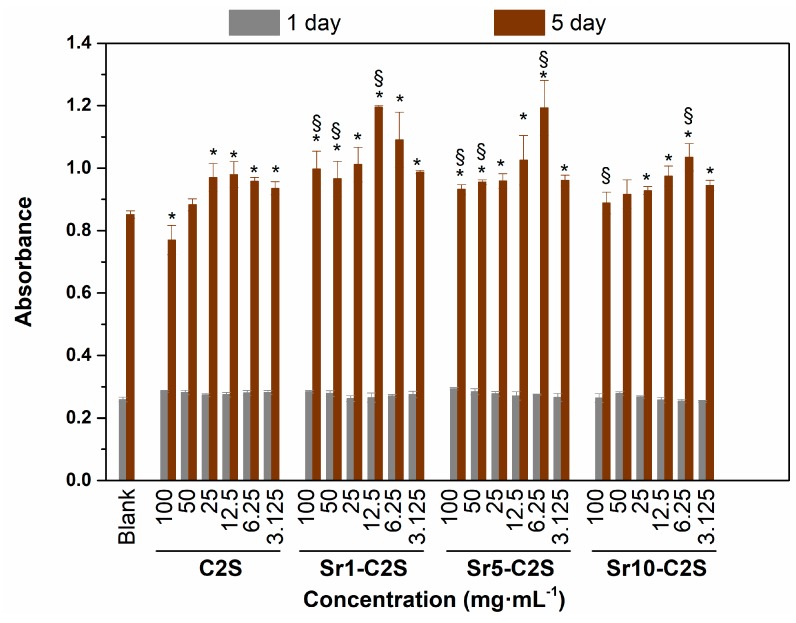
The proliferation of hBMSCs in the medium supplemented with C2S, Sr1-C2S, Sr5-C2S and Sr10-C2S after culturing for 1 and 5 days. The cells cultured in medium without C2S or Sr-C2S were treated as the control group. C2S and Sr-C2S groups show higher proliferation activity of hBMSCs than blank group. Furthermore, Sr-C2S groups show higher proliferation activity of hBMSCs than C2S group. * denotes significant difference exists between this group and the blank group. § denotes significant difference exists between this group and the C2S group at corresponding concentrations.

The effect of C2S and Sr-C2S cements on proliferation of hBMSCs is shown in Figure 11. Compared with the blank control group, both C2S and Sr-C2S bone cements showed a stimulatory effect on hBMSC proliferation at certain concentrations of the extracts, which were 25 to 3.125 mg·mL^−1^ for C2S bone cement, 12.5 to 3.125 mg·mL^−1^ for Sr10-C2S bone cement, and 100 to 3.125 mg·mL^−1^ for Sr1-C2S and Sr5-C2S bone cement, respectively. Furthermore, the cells in Sr-C2S groups showed higher proliferation activity, as compared with that of C2S cement, at 100, 50, and 12.5 mg·mL^−1^ for Sr1-C2S bone cement, 100, 50, and 6.25 mg·mL^−1^ for Sr5-C2S bone cement and 6.25 mg·mL^−1^ for Sr10-C2S bone cement, respectively. Previous studies have shown that Si-containing bioceramics, scaffolds and coatings could improve proliferation and differentiation of osteo-related cells, such as hBMSC, due to promotion effect of Si on osteogenesis [28,29,30,31]. Xing et al. further studied the effects of Si and Sr ions on the proliferation and osteogenic differentiation of hBMSCs and found that Si and Sr ions could synergistically stimulate cell proliferation and osteogenic differentiation of hBMSCs within certain concentrations [21]. The Sr-C2S bone cements in the present study contain both Si and Sr, and it is therefore reasonable to infer that the promoted cell proliferation activity in Sr-C2S group can be due to the synergetic release of Sr and Si ions from the materials. The higher viability of hBMSCs of Sr-C2S group suggests pretty good in vitro cytocompatibility of the bone cements and may lead to an improved osteogenesis ability.

### 3.6. ALP Activity Assay

**Figure 12:**
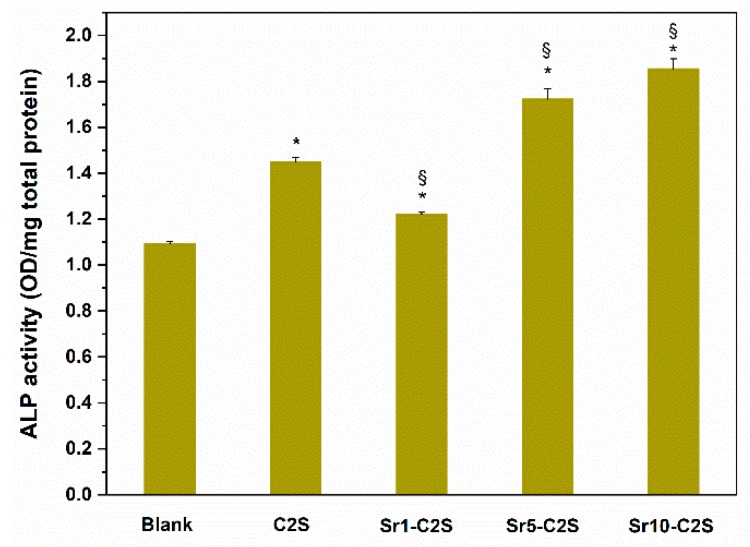
ALP activity of hBMSCs in the medium supplemented with C2S, Sr1-C2S, Sr5-C2S and Sr10-C2S after culturing for 10 days. The cells cultured in medium without C2S or Sr-C2S were treated as the control group. C2S and Sr-C2S groups demonstrate higher ALP activity of hBMSCs than blank group. Furthermore, Sr5-C2S and Sr10-C2S groups demonstrate higher ALP activity of hBMSCs than C2S group. * denotes significant difference exists between this group and the blank group. § denotes significant difference exists between this group and the C2S group.

The ALP activity of the cells in C2S and Sr-C2S groups have a significant (*p* < 0.05) increase compared with the blank control group, which are demonstrated in Figure 12. Moreover, Sr 5-C2S and Sr 10-C2S groups possessed significantly (*p* < 0.05) higher ALP activity than C2S group. Osteogenic differentiation is an important process during bone regeneration and ALP is considered to be an early marker of osteogenic differentiation, which can be used as an indication for evaluating the level of the viability of osteogenic differentiation. In the present study, we found that C2S bone cement can promote osteogenic differentiation of hBMSCs in vitro compared with that of blank group. The combination of Sr element and C2S bone cement can further improve this promotion effect. These results are in good correspondence with those in previous studies, in which it was showed that Si and Sr ions have a synergistic stimulatory effect on the bone-related differentiation of hBMSCs [21]. In the process of bone defects repairing, it is desirable that bone cements could actively induce regeneration of living bone tissues, especially in restoration of sites with large defects and where the bone tissues lack osteogenic capacity, such as osteoporotic bones. The Sr-C2S bone cements in the present study displayed good in vitro ALP activity and might be a potential biomaterial for in situ restoration of bone tissues with poor osteogenesis. Nevertheless, more comprehensive evaluations for different biomarkers in the osteogenic differentiation process, as well as in vivo osteogenesis, should be conducted in future investigation work.

## 4. Conclusions

In the present study, Sr-C2S bone cements with in situ Sr-substitution and homogeneously Sr-distribution was developed for orthopaedic applications for the first time. The Sr-C2S bone cements demonstrated self-setting property and good apatite mineralization ability that are similar to those of C2S bone cements. In addition, Sr-C2S bone cements showed great potential in promoting osteogenesis in a preliminary study on the effects of Sr-C2S bone cements on proliferation and ALP activity of hBMSCs. These results suggest that the Sr-C2S bone cements might be new self-setting materials for osteoporotic bone regeneration and it is worth conduct more intensive studies on their biological performance both in vitro and in vivo.

## Figures and Tables

**Table 1:** Volume of 1 mol·L^−1^ raw reagent solutions (Ca(NO_3_)_2_·4H_2_O, Sr(NO_3_)_2_, Na_2_SiO_3_·9H_2_O and Na_2_CO_3_) for preparation of 0.1 mol C2S, Sr1-C2S, Sr5-C2S and Sr10-C2S.

Unit: mL	C2S	Sr1-C2S	Sr5-C2S	Sr10-C2S
Ca(NO_3_)_2_·4H_2_O	200	198	190	180
Sr(NO_3_)_2_	0	2	10	20
Na_2_SiO_3_·9H_2_O	100	100	100	100
Na_2_CO_3_	100	100	100	100

**Table 2:** Composition of SBF (1000 mL) used in the apatite mineralization test.

**Items**	**NaCl**	**NaHCO_3_**	**KCl**	**K_2_HPO_4_·3H_2_O**	**MgCl_2_·6H_2_O**
**Amount**	8.035 g	0.355 g	0.255 g	0.231 g	0.311 g
**Purity (%)**	99.5	99.5	99.5	99.0	98.0
**Items**	**1.0M-HCl**	**CaCl_2_**	**Na_2_SO_4_**	**Tris**	**1.0M-HCl**
**Amount**	39 mL	0.292 g	0.072 g	6.118 g	0–5 mL
**Purity (%)**	–	95.0	99.0	99.0	–

**Table 3:** 2θ (deg.) values for (−1, 2, 1), (3, 0, 1) and (1, 3, 0) crystallographic plane of C2S and Sr-C2S.

Unit: °	2θ for (−1, 2, 1) ^1^	2θ for (3, 0, 1) ^1^	2θ for (1, 3, 0) ^1^
C2S	32.112	34.305	41.173
Sr1-C2S	32.112	34.302	41.169
Sr5-C2S	32.074	34.227	41.094
Sr10-C2S	32.034	34.148	40.993

^1^ (−1, 2, 1), (3, 0, 1) and (1, 3, 0) are crystal plane indices corresponding to diffraction angles (2θ) listed below them.

**Table 4:** Content of Sr element in Sr-C2S tested by XRF.

Content of Sr		Sr1-C2S	Sr5-C2S	Sr10-C2S
Theoretical content of Sr	mol.%	1	5	10
	wt.%	1.012	4.950	9.642
Actual content of Sr	mol.%	0.300	1.700	6.300
	wt.%	0.330	1.860	6.427
